# COMPARATIVE STUDY OF POSTOPERATIVE PAIN BETWEEN THE LICHTENSTEIN AND LAPAROSCOPY SURGICAL TECHNIQUES FOR THE TREATMENT OF UNILATERAL PRIMARY INGUINAL HERNIA

**DOI:** 10.1590/0102-6720201700030003

**Published:** 2017

**Authors:** Leandro Mendonça PEDROSO, Renato Miranda DE-MELO, Nelson Jorge DA-SILVA-JR

**Affiliations:** 1 Medical, Pharmaceutical and Biomedical Sciences School, Pontifical Catholic University of Goiás (PUC-GO), Goiânia, Goiás, Brazil.

**Keywords:** Pain, postoperative, Visual analog scale, Hernia, inguinal, Laparoscopy

## Abstract

**Background:**

There are several surgical treatment options for inguinal hernia; however, there is no consensus on the literature identifying which surgical technique promotes less postoperative pain.

**Aim:**

To compare the intensity of postoperative pain between the surgical techniques Lichtenstein and transabdominal pre-peritoneal laparoscopy for the treatment of unilateral primary inguinal hernia.

**Methods:**

Were included 60 patients, of which 30 were operated through the Lichtenstein technique and 30 patients through the transabdominal pre-peritoneal laparoscopy. The pain levels were evaluated through the analogue visual scale for 2, 10 and 30 days after the surgery. Additionally, the recurrence rate and the presence of chronic pain and paresthesia were evaluated 12 months after the surgery.

**Results:**

Overall, the data analysis showed significant differences on pain levels between the surgical techniques. There were no significant differences between the pain levels for day 2. However, for 10 and 30 days after the surgery, the pain levels were significantly lower for the patients operated through the transabdominal pre-peritoneal laparoscopy technique compared to the Lichtenstein technique. Furthermore, despite no recurrent hernias for both surgical techniques, 32 % of patients operated through the Lichtenstein technique reported chronic pain and paresthesia 12 months after the surgery, compared with 3,6% of patients operated through the transabdominal pre-peritoneal laparoscopy technique.

**Conclusion:**

There are differences between the surgical techniques, with the transabdominal pre-peritoneal laparoscopy procedure promoting significantly lower postoperative pain (10 and 30 days) and chronic pain (12 months) compared to the Lichtenstein procedure.

## RESUMO

**Racional:**

Existem diversas opções de tratamentos cirúrgicos para hernia inguinal; entretanto, não existe consenso na literatura sobre qual técnica cirúrgica promove menor dor pós-operatória.

**Objetivo:**

Comparar a intensidade de dor pós-operatória entre as técnicas cirúrgicas de Lichtenstein e a laparoscópica pré-peritoneal transabdominal no tratamento da hérnia inguinal unilateral não-recidivada.

**Métodos:**

Foram incluídos 60 pacientes, dos quais 30 foram operados pela técnica de Lichtenstein e 30 pela laparoscópica pré-peritoneal transabdominal. A intensidade da dor foi avaliada por meio da escala visual analógica nos dias 2, 10 e 30 de pós-operatório. A taxa de recidiva, a presença de dor crônica e parestesia local foram avaliadas com 12 meses de pós-operatório.

**Resultados:**

A análise dos dados demonstrou que existem diferenças significantes nos níveis de dor entre as técnicas cirúrgicas. Não houve diferença entre o nível de dor com dois dias. Entretanto, com 10 e 30 dias, o nível de dor foi significativamente menor para os operados pela técnica laparoscópica pré-peritoneal transabdominal, comparado à de Lichtenstein. Além disso, apesar de não haver casos de reincidência para ambas as técnicas, 32% dos pacientes operados por Lichtenstein relataram dor crônica e parestesia local 12 meses depois da operação, comparado a 3,6% da técnica laparoscópica pré-peritoneal transabdominal.

**Conclusão:**

Há diferenças entre as técnicas cirúrgicas, com o procedimento laparoscópico pré-peritoneal transabdominal promovendo níveis de dor pós-operatória (10 e 30 dias) e dor crônica (12 meses) significativamente menores, quando comparado ao procedimento Lichtenstein.

## INTRODUCTION

The surgical treatment of inguinal hernia is one of the most performed medical procedures, with more than 20 million surgeries around the world annually^11^. In Brazil, from 2015 to 2016 the National Health System (Sistema Único de Saúde - SUS) treated more than 117.090 cases of unilateral inguinal hernia through the open technique and 901 cases through laparoscopic techniques^19^. Nowadays, several surgical techniques are available for the treatment of inguinal hernia^5^ ^**,**^ ^10^ ^**,**^ ^15^. However, two techniques are generally accepted as the best treatment options for inguinal hernia repair: the tension-free open Lichtenstein and the laparoscopic procedures^4^. The choice of the more appropriate surgery was based on the rate of recurrent hernia. However, with the technological advances in surgical techniques, the rate of recurrent hernia is now very low regardless of the surgical procedure^7^ ^,^ ^12^. Therefore, other postoperative complications have been used to determine which surgery is more appropriate. 

Postoperative pain is now recognized as one of the major problems related to inguinal hernia repair, as it affects directly the quality of life of patients^8^ ^,^ ^16^. There is, however, a lack of agreement in the literature, as to which inguinal hernia repair technique is the optimum in regards to postoperative pain and complications. There are studies that reported higher postoperative and chronic pain following the open Lichtenstein technique. On the other hand, many studies have described the laparoscopic inguinal hernia repair resulting in a lower incidence of postoperative pain, edema formation and an earlier return to normal activities than the Lichtenstein technique^3^. 

Therefore, the objective of this study was to compare the intensity of postoperative pain between the open Lichtenstein (LC) and transabdominal pre-peritoneal laparoscopy (TAPP) techniques for the treatment of primary inguinal hernia. 

## METHODS

### Study design and participants

This study was approved by the Ethics Committee of the Santa Casa de Misericórdia de Goiânia - GO hospital (Project number 28150514.2.3001.5081) and was performed according to the 466 Resolution. Appropriate informed consent was obtained from all participants.

This was a prospective clinical study to evaluate pain levels following the surgical repair of unilateral primary inguinal hernia. Overall, 60 patients were enrolled. The choice of surgical procedure was randomized, with the first 30 patients evaluated at the Santa Casa da Misericórdia de Goiânia, Brazil, booked through the Municipal Health Office, were operated through the open LC technique and the 30 following patients operated through the TAPP technique. The inclusion criteria were: age between 18-70 years, Goldman surgical risk level I or II and the diagnosis of unilateral primary inguinal hernia. The exclusion criteria were: previous abdominal surgeries, susceptible individuals such as native Brazilians, army and prisoners, not completing the postoperative following at any stage, urgent surgeries and additional surgical procedures such as umbilical hernioraphy, prostatectomy, cholecystectomies and other surgical procedures. Among all patients, two were excluded from the LC group as they did not complete the 12 months postoperative and two from the TAPP group for not completing the 30 days postoperative examination. Data concerning gender, age, body mass index (BMI) and Nyhus classification of the hernia, as well as duration of the surgery, edema occurrence and pain medication use, were recorded for each patient. 

### Surgical procedures

All surgical procedures were performed by the same surgeon. The patients underwent standard routine preoperative examination including. All patients were discharged one day after the surgical procedure, with the exception of one patient that remained in the hospital for two days to drain a scrotal sac seroma. 

#### Lichtenstein technique (LC)

The patient was laid down in the supine position under raquianesthesia. After the asepsis using clorexidine, an oblique incision of approximately 7 cm, on the bisectrix of the angle formed between the inguinal fold and the external edge of the rectus abdominis muscle, with the opening of the pars plana until the inguinal canal. It was dissected laterally until de inguinal arcade and medially until the abdominal rectus. Subsequently, the spermatic funiculus was isolated using Penrose drains number 1, as well as identification, isolation and treatment of the hernia sac. The inguinal canal posterior wall reinforcement was made below and above the internal inguinal ring, using a polypropylene mesh of 12x4 cm, fixed using nylon 2-0 on the pubic tubercle, inguinal arcade and the conjoined tendon. The synthesis of the planes wall was made on the roof of the inguinal canal with nylon 2-0, subcutaneous with regular catgut 3-0 and on the skin using nylon 4-0.

#### Transabdominal pre-peritoneal laparoscopy (TAPP)

For the TAPP procedure, the patient laid down in the Trendelenburg position under general anesthesia. Subsequently, a pneumoperitoneum was created using a Veress needle around the umbilical region inserting three trocars, a 10 mm at umbilical level and two 5 mm at the hemiclavicular level on the left and right sides. An incision was made on the peritoneal membrane above the upper side of the internal ring. The peritoneal membrane was incised just above the superior edge of the inguinal ring and, mobilized laterally until the anterior superior iliac spine, medially until the pubic tubercle, and inferiorly until the ductus deferens. A piece of mesh with 18x12 cm was inserted through the 10 mm trocar and fixed on the superior region of the pubic area, using polyester thread 2-0. Once de mesh was applied, the peritoneum was sewed with the same polyester, in order to induce local reperitonealization.

### Postoperative follow-up

The pain intensity was evaluated through the visual analogue scale (AVS) for three postoperative days: 2, 10 and 30 after the surgery. The recurrence of the hernia, presence of chronic pain and paresthesia were also evaluated 12 months after the surgical procedure.

The information about postoperative pain was recorded by the patient two days after the surgery, and the surgeon performed the 10 and 30 days and 12 months follow-up and the 12 months follow-up after the surgery. The recurrence rate was determined through physical examination and the chronic pain was determined as present or absent, without measuring intensity.

### Statistical analysis

First, was performed a descriptive analysis using the variables gender, age, BMI, Nyhus classification, duration of the surgery, edema occurrence and pain medication use. For the categorical variables, was used the chi-squared test and for the numeric variables the Student´s t-test. A repeated measures ANOVA was used to compare the postoperative pain levels 2, 10 and 30 days after the surgery. The significant interactions were further analyzed using the post-hoc test Tukey´s HSD. For the ANOVA, the assumptions of homogeneity of variance and normality were assessed by scatter plots and normal curves of the residuals, respectively. All statistical analyses were performed using the IBM SPSS Statistics software version 20 (Chicago, USA).

## RESULTS

### Descriptive analysis

Overall, from the total number of 56 patients, 51 were male and five female aged between 26-69 years. There were no significant differences in gender, age, BMI, and duration of the surgery between the patient groups (Table 1). There were, however, significant differences in the occurrence of edema and use of analgesic pain medication, where the LC technique promoted a higher incidence of these variables (Table 1).

**TABLE 1:** Characteristic of patients, surgery and postoperative complications

Characteristics	TAPP	Lichtenstein	p
Age (years)	50.5	59	>0.05
Body mass index (kg/m2)	27.3	25.8	>0.05
Duration of surgery (min)	64	60	>0.05
	Number of patients (%)		
Total	28 (100)	28 (100)	
Male	25 (89.3)	26 (92.9)	>0.05
Female	3 (10.7)	2 (7.1)	>0.05
Side of hernia			
Left	13 (46.4)	15 (53.6)	>0.05
Right	15 (53.6)	13 (46.4)	>0.05
Nyhus Classification			
Type I	1 (3.6)	1 (3.6)	>0.05
Type II	7 (25)	7 (25)	>0.05
Type III			
a	12 (42.8)	11 (39.3)	>0.05
b	8 (28.6)	9 (32.1)	>0.05
c	0	0	
Postoperative complications			
Edema occurrence	6 (8.3)	26 (92.9)	<0.001
Analgesic administration	14 (50)	20 (71.4)	<0.001
Outcomes at 12 months			
Recurrence rate	0	0	
Local paraesthesia	0	9 (32.1)	<0.003
Chronic pain	1 (3.6)	9 (32.1)	<0.003

### Pain levels through the analogue visual scale (AVS)

**FIGURE 1:**
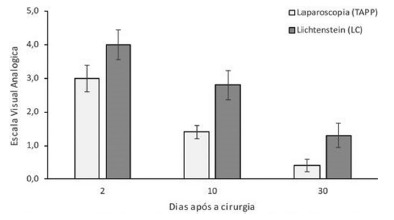
Pain level for LC and TAPP surgical techniques

The ANOVA demonstrated that the patients operated through the TAPP had pain levels significantly lower than the patients operated through the LC procedure (p<0.05, Table 2). There was no significant difference in the pain levels two days postoperative between surgical procedures (p>0.05, Table 3). The mean AVS pain levels were 3±0.4 for the TAPP and 4±0.5 for the LC technique (Figure 1). After 10 days postoperative, the pain levels were significantly lower for the TAPP procedure (1.4±0.2) compared to the LC procedure (2.8±0.4) (p< 0.05, Table 3). Similarly, for the 30 days postoperative, the pain levels were also significantly lower for the TAPP compared to the LC procedure. For the TAPP group the mean AVS pain was 0.4±0.1 and for the LC group was 1.3±0.3 (Figure 1). For the 12 months postoperative follow-up, no patient was diagnosed with recurrent hernia (Table 1). However, the percentage of patients with chronic pain and local paresthesia were higher for the LC group (32 %) compared to the TAPP group (3.6 %,Table 1).

**TABLE 2:** Overall statistical results of the repeated measure ANOVA for the pain levels of patients subjected to inguinal hernia repair through the Lichtenstein (LC) and laparoscopic (TAPP) techniques

Source		df	MS	F	p
Treatment		1	52.952	8.428	0.005
Error		56	6.283		

Bold p values indicates significance

**TABLE 3:** Statistical results of the repeated measures ANOVA for the pain levels for each postoperative period, of patients subjected to inguinal hernia repair through the Lichtenstein (LC) and laparoscopic (TAPP) techniques.

Source	df	MS	F	p
2 days	1	14.414	2.673	0.108
10 days	1	28.677	8.475	0.005
30 days	1	11.918	4.872	0.031

Bold p values indicates significance

## DISCUSSION

Postoperative pain is one of the major factors concerning the choice of a surgical technique for the repair of inguinal hernia. This study determined that patients subjected to the TAPP procedure presented significantly lower levels of postoperative pain compared to the LC, for 10 and 30 days after the surgery. Chronic pain and paresthesia were also present in a lower percentage on patients that underwent the TAPP procedure (3.6%) compared to the LC (32%). Additionally, for the TAPP surgical procedure, the edema incidence and the use of analgesic medication was significantly lower. However, no cases of recurrent hernia were reported for both techniques 12 months after the surgery.

In Brazil, almost 100% of the surgical procedures for inguinal hernia repair from the National Health System (SUS) in 2015/2016 were performed through the open Lichtenstein technique^19^. In addition to the low recurrence rate, the Lichtenstein procedure may be performed in a shorter operating time and cost compared to laparoscopic procedures^4^. There are, however, complications related to the open repair procedures, with postoperative pain regarded as one of the most important. Previous studies reported higher pain level for patients surgically treated with open techniques such as Lichtenstein^13^ ^,^ ^18^. However, a large number of studies evaluated pain 48 h after the surgery, a period which patients are still under the effects of analgesics used on the anesthetics. In this study, the pain levels were also evaluated 10 and 30 days after the surgery, showing higher pain levels on these periods for the LC techniques compared to the TAPP technique. Thus, patients undergoing the laparoscopic procedure, in this and other studies, have reported lower rates of postoperative pain compared to the open techniques.

Chronic pain is defined as an AVS score above zero which lasts for more than three months after a surgical procedure and is described by patients as an ongoing awareness of pain. Chronic pain may be caused by nerve damage during surgery. It may also be related to the positioning of the mesh in the inguinal canal^16^ ^,^ ^17^. In addition to lower short-term postoperative pain, the laparoscopic procedure used in this study promoted significantly lower chronic pain 12 months after the surgery compared to the open LC procedure, 3.6% and 32% respectively. Similarly, in another study, the level of chronic pain was two-times higher for patients that underwent the open Lichtenstein compared to laparoscopic TAPP technique^1^ and was also lower for different laparoscopic techniques such as the total extra-peritoneal repair and transinguinal pre-peritoneal repair^8^ ^,^ ^9^.

The recurrence rate was, for many years, the main factor used to determine the surgical technique to repair inguinal hernias. The incorporation of the tension-free prosthetic mesh by Lichtenstein was the sole responsible for the significant decrease in the rate of recurrent hernia^2^. This technique is considered the “gold standard” for inguinal hernia repair and is currently the reference worldwide. The Lichtenstein method reduced the recurrence inguinal hernia to less than 1%, independently of the level of expertise of the surgeon^3^. In this study, none of the patients presented recurrent hernia 12 months after the surgery. Although 12 months, is a short time for the measurement of recurrence rate, longer term studies have found that the risk of recurrence is similarly low for both techniques after five years of surgery when a proper mesh size is applied^8^ ^,^ ^14^{McCormack, 2005 #11}.

## CONCLUSION

The TAPP technique promoted less postoperative and chronic pain. The laparoscopic procedure TAPP hernia repair was safe and reliable, with a similar recurrence rate to the open Lichtenstein repair. Additionally, the laparoscopic procedure showed clear advantages such as less postoperative and chronic pain, paresthesia and lower incidence of edema and use of pain medication. Therefore, the laparoscopic TAPP procedure should be considered as an appropriate approach for the surgical treatment of unilateral primary inguinal hernia.
